# Supplementary material for: Global, cell non-autonomous gene regulation drives individual lifespan among isogenic C. elegans
Source: eLife. 2021 Feb 1;10:e65026. doi: 10.7554/eLife.65026 (PMC7864635; doi:10.7554/eLife.65026)
Supplement: Supplementary file 1. [file elife-65026-supp1.pdf]

| miRNA | N | *Measure of pixel intensity* | *Time window (hours post-hatch)* | *Slope $R^2$* | *Mean $R^2$* | *Joint $R^2$* | *Joint 95% CI* |
|---|---|---|---|---|---|---|---|
| *let-7* | 283 | 99th percentile | 72–233 | 0.002 | 0.007 | 0.013 | [-0.016, 0.040] |
| *lin-4* | 197 | Mean over 99th percentile | 72–212 | 0.162 | 0.014 | **0.165 (+)** | [0.071, 0.26] |
| *mir-1* | 72 | Maximum (head) | 72–192 | 0.086 | 0.002 | 0.091 | [-0.035, 0.19] |
| *mir-47* | 408 | Maximum | 72–201 | 0.257 | 0.104 | **0.287 (+)** | [0.22, 0.35] |
| *mir-51* | 83 | Maximum | 72–236 | 0.002 | 0.015 | 0.015 | [-0.067, 0.11] |
| *mir-59* | 104 | 95th percentile (vulva) | 72–150 | 0.044 | 0.031 | 0.067 | [-0.037, 0.14] |
| *mir-60* | 218 | 99th percentile | 72–210 | 0.117 | 0.101 | **0.174 (+)** | [0.061, 0.27] |
| *mir-63* | 138 | 99th percentile | 72–231 | 0.121 | 0.025 | 0.138 | [0.036, 0.24] |
| *mir-79* | 86 | Maximum | 72–120 | 0.035 | 0.002 | 0.038 | [-0.043, 0.11] |
| *mir-84* | 142 | 99th percentile | 72–231 | 0.006 | 0.059 | 0.059 | [-0.04, 0.15] |
| *mir-85* | 184 | 99th percentile | 72–172 | 0.177 | 0.041 | **0.177 (+)** | [0.10, 0.26] |
| *mir-90* | 173 | 95th percentile | 72–221 | 0.101 | 0.142 | **0.172 (+)** | [0.076, 0.29] |
| *mir-228* | 151 | Mean over 99th percentile | 72–170 | 0.122 | 0.019 | 0.125 | [0.039, 0.22] |
| *mir-240-786* | 160 | 95th percentile | 72–228 | 0.178 | 0.187 | **0.232 (-)** | [0.13, 0.33] |
| *mir-241* | 97 | 99th percentile (head) | 72–120 | 0.023 | 0.013 | 0.039 | [-0.035, 0.10] |
| *mir-242* | 98 | Maximum | 72–204 | 0.004 | 0.031 | 0.037 | [-0.040, 0.11] |
| *mir-243* | 343 | Mean | 72–176 | 0.305 | 0.091 | **0.326 (+)** | [0.25, 0.40] |
| *mir-246* | 193 | 95th percentile | 72–200 | 0.120 | 0.024 | 0.128 | [0.053, 0.20] |
| *mir-360* | 87 | Maximum | 72–191 | 0.018 | 0.018 | 0.027 | [-0.044, 0.093] |
| *mir-788* | 187 | Maximum | 72–96 | 0.010 | 0.003 | 0.010 | [-0.023, 0.039] |
| *mir-793* | 177 | Maximum | 72–237 | 0.158 | 0.046 | **0.169 (+)** | [0.079, 0.26] |
| *mir-794* | 91 | Maximum | 72–96 | 0.001 | 0.055 | 0.058 | [-0.045, 0.15] |

**Table S1:** Fixed-time-window correlation of *PmiRNA*::GFP reporter expression with lifespan. A joint regression of slope and average expression against lifespan was performed using a window starting at 72 hours post-hatch and ending at the 90% survival timepoint. $R^2$ values exceeding 0.15 are indicated by **bolded** text. The 95% confidence interval (CI) for each joint correlation coefficient is also shown. The direction of correlation, derived from the individual regression on slope and mean expression (which we observed to always correlate in the same direction), is indicated by (+) or (-), respectively.

| *miRNA* | *N* | *Measure of pixel intensity* | *Time window (days post-hatch)* | *Slope* $R^2$ | *Mean* $R^2$ | *Joint* $R^2$ | *Joint 95% CI* |
|---|---|---|---|---|---|---|---|
| *let-7* | 307 | 99th percentile | 6.0–7.5 | 0.014 | 0.024 | 0.036 | [-0.007, 0.081] |
| *lin-4* | 209 | Mean over 99th percentile | 4.5–8.0 | 0.200 | 0.021 | **0.203 (+)** | [0.114, 0.293] |
| *mir-1* | 79 | Maximum (head) | 6.0–7.5 | 0.102 | 0.025 | 0.117 | [0.009, 0.237] |
| *mir-47* | 433 | Maximum | 5.0–8.0 | 0.125 | 0.165 | **0.290 (+)** | [0.219, 0.363] |
| *mir-51* | 87 | Maximum | 8.0–8.5 | 0.025 | 0.048 | 0.098 | [-0.016, 0.210] |
| *mir-59* | 104 | 95th percentile (vulva) | 4.5–6.5 | 0.002 | 0.092 | 0.092 | [-0.017, 0.187] |
| *mir-60* | 227 | 99th percentile | 7.5–8.0 | 0.017 | 0.177 | **0.189 (+)** | [0.073, 0.302] |
| *mir-63* | 147 | 99th percentile | 3.0–9.0 | 0.109 | 0.019 | 0.124 | [0.033, 0.219] |
| *mir-79* | 86 | Maximum | 3.5–4.0 | 0.005 | 0.010 | 0.025 | [-0.033, 0.076] |
| *mir-84* | 157 | 99th percentile | 3.0–7.5 | 0.024 | 0.023 | 0.091 | [-0.005, 0.172] |
| *mir-85* | 194 | 99th percentile | 4.0–6.5 | 0.115 | 0.048 | 0.146 | [0.065, 0.228] |
| *mir-90* | 181 | 95th percentile | 8.0–8.5 | 0.014 | 0.215 | **0.229 (+)** | [0.120, 0.347] |
| *mir-228* | 164 | Mean over 99th percentile | 5.5–6.5 | 0.059 | 0.059 | 0.110 | [0.036, 0.193] |
| *mir-240-786* | 172 | 95th percentile | 7.5–8.5 | 0.043 | 0.268 | **0.291 (-)** | [0.167, 0.415] |
| *mir-241* | 97 | 99th percentile (head) | 3.0–3.5 | 0.013 | 0.037 | 0.051 | [-0.040, 0.142] |
| *mir-242* | 107 | Maximum | 4.5–5.5 | 0.096 | 0.012 | 0.096 | [-0.008, 0.192] |
| *mir-243* | 360 | Mean | 4.5–7.0 | 0.203 | 0.160 | **0.325 (+)** | [0.255, 0.399] |
| *mir-246* | 205 | 95th percentile | 7.0–7.5 | 0.025 | 0.134 | **0.181 (+)** | [0.087, 0.277] |
| *mir-360* | 87 | Maximum | 4.0–5.0 | 0.061 | 0.009 | 0.071 | [-0.025, 0.160] |
| *mir-788* | 187 | Maximum | 3.0–3.5 | 0.000 | 0.005 | 0.006 | [-0.029, 0.037] |
| *mir-793* | 186 | Maximum | 5.5–9.5 | 0.120 | 0.070 | **0.161 (+)** | [0.074, 0.243] |
| *mir-794* | 91 | Maximum | 3.0–3.5 | 0.009 | 0.082 | 0.093 | [-0.032, 0.209] |

**Table S2:** Correlation of *PmiRNA*::GFP reporters with lifespan using a sliding window and 95% survival endpoint. A joint regression of slope and average expression against lifespan was performed using a sliding time window (minimum width of 12 hours) beginning at 3 days post-hatch and ending at the 95% survival timepoint. The optimal time window and highest correlation achieved is reported. $R^2$ values exceeding 0.15 are indicated by **bolded** text. The 95% confidence interval (CI) for each joint correlation coefficient is also shown. The direction of correlation, derived from the individual regression on slope and mean expression (which we observed to always correlate in the same direction), is indicated by (+) or (-), respectively.

| *Reporter* | *N* | *Measure of pixel intensity* | *Time window (hours post-hatch)* | *Slope $R^2$* | *Mean $R^2$* | *Joint $R^2$* | *Joint 95% CI* |
|---|---|---|---|---|---|---|---|
| *cpna-2* | 65 | 99th percentile | 168–192 (S) | 0.112 | 0.006 | 0.123 | [0.007, 0.242] |
| *cpna-2* | 64 | 99th percentile | 72–196 (F) | 0.007 | 0.017 | 0.018 | [-0.081, 0.093] |
| *myo-2* | 171 | Maximum | 108–156 (S) | 0.107 | 0.005 | 0.112 | [0.024, 0.191] |
| *myo-2* | 163 | Maximum | 72–168 (F) | 0.069 | 0.009 | 0.071 | [0.004, 0.144] |

**Table S3:** Correlation with lifespan of promoter::GFP reporters for two non-miRNA promoters. A joint regression on slope and average expression against lifespan was performed using a sliding 12-hour window beginning at 3 days post-hatch (indicated by 'S'). The optimal time window and highest correlation achieved is reported as the joint regression $R^2$. Results from using a fixed window from 3 days post-hatch to the 90% survival timepoint (indicated by 'F') are also shown.

| *miRNA* | *N* | *Measure of pixel intensity* | *Time window (hours post-hatch)* | *Maximal expression $R^2$* | *p-value* | *Day of max expression $R^2$* | *p-value* |
|---|---|---|---|---|---|---|---|
| *let-7* | 283 | 99th percentile | 72–233 | 0.000 | 0.989 | 0.001 | 0.681 |
| *lin-4* | 197 | Mean over 99th percentile | 72–212 | 0.000 | 0.853 | 0.001 | 0.638 |
| *mir-1* | 72 | Maximum (head) | 72–192 | 0.012 | 0.307 | 0.010 | 0.353 |
| *mir-47* | 408 | Maximum | 72–201 | 0.000 | 0.779 | 0.020 | 0.004 |
| *mir-51* | 83 | Maximum | 72–236 | 0.030 | 0.116 | 0.001 | 0.798 |
| *mir-59* | 104 | 95th percentile (vulva) | 72–150 | 0.000 | 0.912 | 0.002 | 0.670 |
| *mir-60* | 218 | 99th percentile | 72–210 | 0.003 | 0.425 | 0.025 | 0.019 |
| *mir-63* | 138 | 99th percentile | 72–231 | 0.002 | 0.640 | 0.000 | 0.956 |
| *mir-79* | 86 | Maximum | 72–120 | 0.020 | 0.190 | 0.008 | 0.402 |
| *mir-84* | 142 | 99th percentile | 72–231 | 0.005 | 0.397 | 0.005 | 0.388 |
| *mir-85* | 184 | 99th percentile | 72–172 | 0.010 | 0.169 | 0.070 | 0.000 |
| *mir-90* | 173 | 95th percentile | 72–221 | 0.013 | 0.138 | 0.028 | 0.028 |
| *mir-228* | 151 | Mean over 99th percentile | 72–170 | 0.022 | 0.071 | 0.027 | 0.044 |
| *mir-240-786* | 160 | 95th percentile | 72–228 | 0.122 | 0.000 | 0.030 | 0.028 |
| *mir-241* | 97 | 99th percentile (head) | 72–120 | 0.009 | 0.347 | 0.000 | 0.905 |
| *mir-242* | 98 | Maximum | 72–204 | 0.002 | 0.669 | 0.000 | 0.878 |
| *mir-243* | 343 | Mean | 72–176 | 0.001 | 0.534 | 0.028 | 0.002 |
| *mir-246* | 193 | 95th percentile | 72–200 | 0.011 | 0.142 | 0.042 | 0.004 |
| *mir-360* | 87 | Maximum | 72–191 | 0.000 | 0.916 | 0.007 | 0.438 |
| *mir-788* | 187 | Maximum | 72–96 | 0.006 | 0.299 | 0.001 | 0.682 |
| *mir-793* | 177 | Maximum | 72–237 | 0.004 | 0.400 | 0.002 | 0.517 |
| *mir-794* | 91 | Maximum | 72–96 | 0.050 | 0.032 | 0.000 | 0.855 |

**Table S4:** Correlation coefficients and p-values for regression of lifespan against the whole-life maximum value of *PmiRNA*::GFP expression (measured as noted), or the day that that maximum value was achieved.

| *miRNA* | *N* | *Time window (hours post-hatch)* | *GFP slope $R^2$* | *GFP mean $R^2$* | *AF slope $R^2$* | *AF mean $R^2$* | *Summed $R^2$* | *Joint $R^2$* |
|---|---|---|---|---|---|---|---|---|
| *lin-4* | 197 | 72–212 | 0.162 | 0.014 | 0.140 | 0.037 | 0.353 | 0.302 |
| *mir-47* | 408 | 72–201 | 0.257 | 0.104 | 0.062 | 0.030 | 0.453 | 0.313 |
| *mir-60* | 218 | 72–210 | 0.117 | 0.101 | 0.145 | 0.059 | 0.421 | 0.241 |
| *mir-90* | 184 | 72–172 | 0.101 | 0.142 | 0.076 | 0.084 | 0.403 | 0.304 |
| *mir-228* | 173 | 72–221 | 0.122 | 0.019 | 0.090 | 0.100 | 0.331 | 0.204 |
| *mir-240-786* | 160 | 72–228 | 0.178 | 0.187 | 0.009 | 0.031 | 0.405 | 0.237 |
| *mir-243* | 343 | 72–176 | 0.305 | 0.091 | 0.216 | 0.060 | 0.672 | 0.408 |
| *mir-246* | 193 | 72–200 | 0.120 | 0.024 | 0.101 | 0.058 | 0.303 | 0.191 |
| *mir-793* | 177 | 72–237 | 0.158 | 0.046 | 0.094 | 0.043 | 0.341 | 0.258 |

**Table S5:** Correlation coefficients for *PmiRNA*::GFP (slope and average), autofluorescence (slope and average of 95th percentile intensity), and joint *PmiRNA*::GFP and autofluorescence regression against lifespan.

| Experiment | N | Measure of pixel intensity | Time window (hours post-hatch) | Slope $R^2$ | Mean $R^2$ | Joint $R^2$ | Joint 95% CI |
|---|---|---|---|---|---|---|---|
| *let-7_20170526* | 101 | 99th percentile | 72–213 | 0.004 | 0.001 | 0.004 | [-0.029, 0.047] |
| *let-7_20170517* | 100 | 99th percentile | 72–208 | 0.042 | 0.001 | 0.045 | [-0.048, 0.127] |
| *let-7_20170216* | 48 | 99th percentile | 72–217 | 0.005 | 0.047 | 0.320 | [0.148, 0.520] |
| *let-7_20170208* | 31 | 99th percentile | 72–182 | 0.078 | 0.401 | 0.401 | [0.180,0.691] |
| *lin-4_20171222* | 99 | Mean over 99th percentile | 72–209 | 0.075 | 0.013 | 0.124 | [-0.024, 0.275] |
| *lin-4_20171219* | 97 | Mean over 99th percentile | 72–214 | 0.152 | 0.004 | 0.152 | [-0.034, 0.133] |
| *mir-1_20170518* | 72 | Maximum (head) | 72–192 | 0.084 | 0.002 | 0.086 | [-0.026, 0.182] |
| *mir-47_20190213* | 74 | Maximum | 72–185 | 0.041 | 0.205 | 0.261 | [0.137, 0.416] |
| *mir-47_20181101* | 79 | Maximum | 72–196 | 0.246 | 0.203 | 0.404 | [0.293, 0.547] |
| *mir-47_20180511* | 89 | Maximum | 72–196 | 0.208 | 0.082 | 0.234 | [0.072, 0.416] |
| *mir-47_20170728* | 96 | Maximum | 72–201 | 0.196 | 0.127 | 0.419 | [0.294, 0.562] |
| *mir-47_20170504* | 71 | Maximum | 72–213 | 0.219 | 0.000 | 0.226 | [0.066, 0.394] |
| *mir-51_20180530* | 83 | Maximum | 72–236 | 0.004 | 0.016 | 0.016 | [-0.068, 0.110] |
| *mir-59_20170811* | 104 | 95th percentile (vulva) | 72–144 | 0.026 | 0.026 | 0.064 | [-0.031, 0.137] |
| *mir-60_20180917* | 109 | 99th percentile | 72–204 | 0.064 | 0.101 | 0.145 | [-0.007, 0.277] |
| *mir-60_20180518* | 111 | 99th percentile | 72–208 | 0.184 | 0.114 | 0.249 | [0.135, 0.386] |
| *mir-63_20161215* | 62 | 99th percentile | 72–245 | 0.061 | 0.004 | 0.068 | [-0.077, 0.199] |
| *mir-63_20161202* | 75 | 99th percentile | 72–229 | 0.019 | 0.029 | 0.249 | [0.088, 0.429] |
| *mir-79_20171027* | 86 | Maximum | 72–120 | 0.034 | 0.001 | 0.034 | [-0.045, 0.097] |
| *mir-84_20181018* | 77 | 99th percentile | 72–214 | 0.015 | 0.012 | 0.015 | [-0.077, 0.096] |
| *mir-84_20180928* | 65 | 99th percentile | 72–236 | 0.045 | 0.073 | 0.115 | [-0.028, 0.307] |
| *mir-85_20180914* | 87 | 99th percentile | 72–207 | 0.138 | 0.040 | 0.145 | [0.025, 0.260] |
| *mir-85_20180525* | 97 | 99th percentile | 72–181 | 0.214 | 0.066 | 0.220 | [0.083, 0.362] |
| *mir-90_20190430* | 89 | 95th percentile | 72–212 | 0.027 | 0.151 | 0.177 | [0.045, 0.333] |
| *mir-90_20180928* | 80 | 95th percentile | 72–218 | 0.087 | 0.070 | 0.114 | [-0.031, 0.248] |
| *mir-228_20180914* | 73 | Mean over 99th percentile | 72–218 | 0.125 | 0.008 | 0.139 | [-0.001, 0.288] |

| | | | | | | | |
|---|---|---|---|---|---|---|---|
| *mir-228_20180504* | 77 | Mean over 99th percentile | 72–176 | 0.223 | 0.048 | 0.223 | [0.046, 0.393] |
| *mir-240-786_20181004* | 100 | 95th percentile | 72–228 | 0.219 | 0.242 | 0.258 | [0.127, 0.399] |
| *mir-240-786_20181128* | 60 | 95th percentile | 72–212 | 0.234 | 0.160 | 0.237 | [0.065, 0.435] |
| *mir-241_20170809* | 97 | 99th percentile (head) | 72–120 | 0.027 | 0.013 | 0.039 | [-0.048, 0.103] |
| *mir-242_20181105* | 98 | Maximum | 72–204 | 0.012 | 0.034 | 0.036 | [-0.047, 0.103] |
| *mir-243_20190122* | 90 | Mean | 72–184 | 0.453 | 0.084 | 0.479 | [0.341, 0.631] |
| *mir-243_20181107* | 109 | Mean | 72–200 | 0.193 | 0.068 | 0.255 | [0.116, 0.388] |
| *mir-243_20180613* | 60 | Mean | 72–166 | 0.342 | 0.001 | 0.363 | [0.210, 0.524] |
| *mir-243_20171128* | 82 | Mean | 72–181 | 0.244 | 0.286 | 0.331 | [0.160, 0.554] |
| *mir-246_20180612* | 100 | 95th percentile | 72–184 | 0.214 | 0.101 | 0.223 | [0.100, 0.366] |
| *mir-246_20171116* | 93 | 95th percentile | 72–209 | 0.091 | 0.039 | 0.104 | [0.007, 0.201] |
| *mir-360_20180418* | 87 | Maximum | 72–191 | 0.018 | 0.018 | 0.027 | [-0.051, 0.103] |
| *mir-788_20180427* | 100 | Maximum | 72–96 | 0.018 | 0.005 | 0.018 | [-0.035, 0.072] |
| *mir-788_20171027* | 87 | Maximum | 72–96 | 0.030 | 0.011 | 0.041 | [-0.048, 0.123] |
| *mir-793_20190606* | 79 | Maximum | 72–235 | 0.084 | 0.026 | 0.119 | [0.005, 0.235] |
| *mir-793_20181121* | 97 | Maximum | 72–238 | 0.205 | 0.063 | 0.219 | [0.058, 0.375] |
| *mir-794_20190320* | 91 | Maximum | 72–96 | 0.005 | 0.057 | 0.059 | [-0.044, 0.153] |

**Table S6:** Regression results from individual biological replicates using raw data. A joint regression on slope and average expression against lifespan was performed using a window starting at 72 hours post-hatch and ending at the 90% survival timepoint.
